# Oligometastatic Disease in Non-Small-Cell Lung Cancer: An Update

**DOI:** 10.3390/cancers14051350

**Published:** 2022-03-06

**Authors:** Yi-Hsing Chen, Ue-Cheung Ho, Lu-Ting Kuo

**Affiliations:** 1 Division of Neurosurgery, Department of Surgery, National Taiwan University Hospital Yunlin Branch, Douliu 640, Taiwan; b92401054@ntu.edu.tw (Y.-H.C.); ycho0510@ntuh.gov.tw (U.-C.H.); 2 Division of Neurosurgery, Department of Surgery, National Taiwan University Hospital, Taipei 100, Taiwan

**Keywords:** local therapy, oligometastases, non-small-cell lung cancer, surgery

## Abstract

**Simple Summary:**

Approximately 7–50% of patients with non-small-cell lung cancer (NSCLC) develop oligometastases, which are new tumors found in another part of the body, arising from cancer cells of the original tumor that have travelled through the body. In recent years, these patients have been increasingly regarded as a distinct group that could benefit from treatment that intends to cure the disease, rather than palliative care, to achieve a better clinical outcome. Various treatment procedures have been developed for treating NSCLC patients with different oligometastatic sites. In addition, the newly proposed uniform definition for oligometastases as well as ongoing trials may lead to increased appropriate patient selection and evaluation of treatment effectiveness. The aim of this review article is to summarize the latest evidence regarding optimal management strategies for NSCLC patients with oligometastases.

**Abstract:**

Oligometastatic non-small-cell lung cancer (NSCLC) is a distinct entity that is different from localized and disseminated diseases. The definition of oligometastatic NSCLC varies across studies in past decades owing to the use of different imaging modalities; however, a uniform definition of oligometastatic NSCLC has been proposed, and this may facilitate trial design and evaluation of certain interventions. Patients with oligometastatic NSCLC are candidates for curative-intent management, in which local ablative treatment, such as surgery or stereotactic radiosurgery, should be instituted to improve clinical outcomes. Although current guidelines recommend that local therapy for thoracic and metastatic lesions should be considered for patients with oligometastatic NSCLC with stable disease after systemic therapy, optimal management strategies for different oligometastatic sites have not been established. Additionally, the development of personalized therapies for individual patients with oligometastatic NSCLC to improve their quality of life and overall survival should also be addressed. Here, we review relevant articles on the management of patients with oligometastatic NSCLC and categorize the disease according to the site of metastases. Ongoing trials are also summarized to determine future directions and expectations for new treatment modalities to improve patient management.

## 1. Introduction

Lung cancer is one of the most common cancer types worldwide [1,2,3,4], and non-small-cell lung cancer (NSCLC) is the most frequently diagnosed subtype [1,3,5,6,7]. With advancements in diagnostic tools and the establishment of screening programs, more new cases of lung cancer are diagnosed each year. However, approximately 40–70% of newly diagnosed cases are first identified at an advanced stage [5,6,8,9]. The management of patients with advanced NSCLC should be individualized according to histological type, mutation profile, systemic disease burden, and general performance status. Improving our understanding of tumor biology and molecular testing could facilitate the establishment of optimal therapies [10,11]. Approximately 70% of patients with advanced NSCLC harbor actionable mutation targets, and approximately 30% of these patients could benefit from targeted therapy [5,12,13]. For patients with advanced NSCLC and no targetable gene alterations, platinum-based doublet chemotherapy remains the standard first-line treatment [1,2,7]. Despite the recent progress in the management of advanced NSCLC, the prognosis of these patients remains poor, and the reported median overall survival is only 8–12 months [1,7,14]. By contrast, in patients with targetable gene alterations [15,16,17,18,19], targeted therapies have improved the median overall survival to 26–46 month. Continuing clinical trials have aimed to develop new drugs, including targeted therapies and immunotherapies, and these approaches have been broadly applied to improve the management of advanced NSCLC.

Modern imaging techniques, such as magnetic resonance imaging (MRI) and positron emission tomography (PET), have enabled accurate evaluation of the location and extent of metastatic disease. Comprehensive investigations during NSCLC staging are essential because treatment modalities and clinical outcomes vary considerably according to the extent of systemic disease burden [1]. The disease in patients with advanced NSCLC is generally considered incurable, and surgical excision has no effect on progression-free survival (PFS) or overall survival (OS). However, several studies have reported promising outcomes of local treatment, including surgery or radiotherapy, for patients with limited metastases or recurrences [20,21,22,23,24,25,26,27,28]. Oligometastases were first described in 1995 and were considered distinct disease entities with distant metastases in limited regions [25]. Patients with oligometastases may receive definite curative treatment, whereas patients with more aggressive disease typically only benefit from systemic therapy [7]. The exact number of metastasis-defining oligometastases has not been established; however, most reported studies did not include patients with more than five metastases [5,7,29,30,31]. Interestingly, most studies on oligometastases in NSCLC have not provided a clear definition of the disease [32]. Moreover, a recent systemic review on the definition of synchronous oligometastatic NSCLC reported that a uniform definition of synchronous oligometastatic NSCLC does not exist [32]. The number of involved locations and the number of metastases at a specific location have also not been defined in the literature. Recently, a consensus report on the definition of synchronous oligometastatic NSCLC was proposed [33]; in this report, the authors defined synchronous oligometastases as having no more than five metastases and three involved organs, and mediastinal lymph nodes were not considered metastatic sites. Furthermore, they reported that PET and brain MRI were required for staging. Thus, with the availability of well-established diagnostic protocols and imaging modalities, it is important to adopt a uniform definition of oligometastatic NSCLC in order to develop appropriate patient selection methods for clinical trials in the future. Approximately 7–50% of patients with NSCLC present with oligometastases, depending on the inclusion criteria [7,34]. Previous retrospective studies have reported promising outcomes of local treatment in patients with NSCLC having different oligometastatic sites [21,22,26]; however, the observed improvements in survival may have only reflected selection bias rather than treatment effects [35]. Thus, a meta-analysis was conducted to investigate the prognostic factors of synchronous oligometastatic NSCLC, and the authors found that aggressive local treatment was a positive prognostic factor [5]. Recently, a multicenter randomized trial also reported that local consolidative therapy (LCT), including radiotherapy or surgery, improves median PFS in patients with oligometastatic NSCLC after first-line systemic therapy [36]. However, most patients in the randomized trial did not harbor targetable gene alterations. Whether LCT is beneficial for patients with oligometastatic NSCLC after targeted therapy should be elucidated, and further studies are currently underway (ClinicalTrials.gov identifier: NCT03410043).

In this review, we discuss recent advancements in the management of patients with oligometastatic NSCLC and the categorization of oligometastatic NSCLC according to the site of metastases.

## 2. Brain Oligometastases

Approximately 25–30% of patients with newly diagnosed stage IV NSCLC have brain metastases [7,37]. The median OS of patients with NSCLC with brain metastases is less than 6 months when their intracranial pathologies are treated with steroids or whole brain radiotherapy (WBRT) alone [38,39,40]. Surgical resection of brain metastases is indicated if the lesions cause neurological symptoms or increased intracranial pressure; otherwise, systemic therapy should be prescribed at first [41]. Regarding local control after surgery, postoperative WBRT has been the standard treatment for patients with up to four brain metastases [42,43,44]. However, neurocognitive toxicity due to WBRT is a major concern, and this approach is therefore not recommended in patients with good performance status because the addition of WBRT only improves PFS, not OS. SRS could be an alternative modality to WBRT for treating patients with NSCLC with brain oligometastases after metastasectomy without compromising the recurrence rate [43].

The use of LCT in patients with NSCLC with oligometastases at sites other than the brain has not been extensively studied; however, LCT has been thoroughly evaluated and implemented in the treatment of patients with NSCLC with brain oligometastases. The option of LCT in these patients includes surgery and stereotactic radiosurgery (SRS). Based on the concept of oligometastases and careful selection of patients, patients with limited brain metastases could benefit from LCT and show increased median OS (7–31 months) [23,45,46,47,48,49,50]. However, no prospective randomized trials have directly compared surgery with SRS in this group. One meta-analysis proposed a risk classification to stratify patients with NSCLC with brain oligometastases who underwent LCT and found that patients presenting with metachronous metastases and negative intrathoracic nodal involvement were most likely to have longer median OS [49]. However, one retrospective study reported improved median OS in patients with NSCLC patients with synchronous brain oligometastases who received LCT regardless of nodal status [50]. In addition, similar OS benefits of LCT were observed in both NSCLC patients with synchronous or metachronous brain oligometastases [46,51]. Recent guidelines have also suggested that LCT should be considered in NSCLC patients with synchronous or metachronous brain metastasis [37]. These inconsistent results reflect the retrospective nature of the studies, various definitions of oligometastases, and multiple treatment modalities. This highlights the importance of controlling these factors for the design of future clinical trials. Despite these study limitations, definitive LCT should be considered in patients in whom such treatment could be safely and effectively instituted to achieve long-term survival. The updated National Comprehensive Cancer Network guidelines also recommend that local therapy for thoracic and metastatic lesions should be considered for patients with oligometastatic NSCLC with stable disease after systemic therapy [41]. More studies have explored subgroups of patients with oligometastatic NSCLC who exhibit promising clinical outcomes after LCT [49,52,53,54,55,56]. These efforts could facilitate accurate selection of patients who would most benefit from LCT and guide clinical decisions and the design of prospective clinical trials in the future.

In the era of targeted therapy and immunotherapy, significantly prolonged survival has been observed in patients harboring targetable gene alterations. However, it is unclear whether LCT has beneficial effects in patients with NSCLC with brain oligometastases receiving first-line novel therapies. In addition, studies are currently evaluating other modalities for the treatment of patients with NSCLC with brain oligometastases (Table 1). We expect more consolidated evidence and more treatment options for patients with NSCLC with brain oligometastases in the future.

## 3. Adrenal Oligometastases

Less than 10% of patients with NSCLC have adrenal gland metastases at the time of initial diagnosis [57]. Surgical excision, radiation therapy, and ablative therapy can be applied as treatment options. Several studies have reported that adrenalectomy improves survival in patients with metastatic NSCLC [58,59,60,61,62]. Importantly, either open or laparoscopic adrenalectomy can be performed in these patients, and the median OS increases if radical treatment can be achieved. Drake et al. analyzed the benefits of laparoscopic adrenalectomy in malignant metastases [63] and retrospectively reviewed a prospectively maintained database of adrenalectomies for metastases from 1995 to 2016. In total, 59 of 62 patients underwent laparoscopic surgery, and patients with NSCLC accounted for 33% of all patients. Outcomes were similar in open surgery when performed by experienced surgeons. Compared with those who do not undergo surgery, selected patients who underwent laparoscopic adrenalectomy exhibited prolonged survival. Factors such as lack of mediastinal lymph node invasion, stable primary tumor, and longer disease-free interval were better predictors of survival in patients who underwent adrenalectomy. A review published by Gao et al. described a pooled analysis of 98 patients with isolated adrenal metastasis and NSCLC and demonstrated that patients with metachronous metastasis had significantly longer survival than patients with synchronous metastasis (median OS: 34 months versus 12 months). In addition, patients with adrenal metastasis negative for lymph node metastasis had significantly better outcomes than those who were positive for lymph node metastasis (median OS: 40 months versus 13 months) [64].

Minimal invasive ablation therapy can be applied to patients who cannot tolerate surgery. Botsa et al. showed that radiofrequency ablation (RFA) and microwave ablation (MWA) were effective and safe methods, particularly in patients with adrenal tumors less than 3.5 cm in diameter [65]. In this 5-year single-center study, 71 patients were enrolled and underwent RFA and MWA. The local recurrence rate in the RFA group was 17.1%, compared with 19.4% in the MWA group. The local recurrence rate was significantly higher for those with tumor sizes greater than 3.5 cm than for those with tumors 3.5 cm or smaller (65.2% versus 16.7%, respectively). There were no significant differences in the median survival time between the RFA and MWA groups (14.0 months versus 14.6 months, respectively). By contrast, other studies have shown that tumor size is a significant parameter predicting treatment outcomes following RFA [66,67].

In addition to RFA and MWA, other modalities of LCT include surgery and radiotherapy. In a multicenter, randomized, phase II trial enrolling patients with stage IV NSCLC including adrenal oligometastases, patients in the LCT arm receiving LCT immediately after front-line systemic therapy showed significantly improved median PFS compared with those who received maintenance therapy or observation [36]. A single-center clinical trial carried out in 2020 showed that stereotactic body radiotherapy (SBRT), which was delivered to a biological effect dose of 75 gray (Gy), in 28 adrenal metastases (46% from NSCLC) yielded promising local control rates [68], with complete response in 29% of lesions and partial response in 57% of lesions. These observations demonstrated the superior trend in treatment response in groups with a higher biologically effective dose (BED; ≥75 Gy); further studies are needed to confirm these results. Notably, by analyzing tumors based on volume, other studies have also reported comparable control rates for adrenal metastases by SBRT [69,70]. Some ongoing trials are current testing the effects of SBRT on oligometastatic NSCLC including adrenal oligometastases (Table 2).

## 4. Bone Oligometastases

Among patients with lung cancer, 30–40% present with bone metastases in advanced-stage disease, with the ribs being the most common metastatic site [71,72,73]. The median survival of patients with lung cancer after bone metastases is usually less than 1 year [71,72,73,74,75,76,77]. Bone diseases are often complicated by skeletal-related events (SREs), including pathological fractures, spinal cord compression, skeletal pain, and hypercalcemia, which significantly decrease patients’ quality of life in terms of physical, functional, and emotional well-being. In a multivariate analysis of prognostic factors in patients with NSCLC with bone metastases [78], Wu et al. identified multiple bone metastases, high serum alkaline phosphatase, lactate dehydrogenase, and high Eastern Cooperative Oncology Group performance status as negative prognostic factors. Currently, in patients with NSCLC with bone metastases, palliative radiotherapy and systemic therapy, including bisphosphonates and other bone-targeted agents (BTAs), are standard therapeutic choices [79]. Bone metastases develop via osteolytic or osteoblastic mechanisms, which involve different molecular pathways and cells [80,81], and these fundamental mechanisms are targeted by BTAs in the treatment of NSCLC with bone metastases and SREs [81]. The role of the nuclear factor-kappa B (NF-κB) signaling pathway in the pathogenesis of NSCLC has been elucidated in animal models, and both receptor activator of nuclear factor-kappa (RANK) and NF-κB function to promote cancer with bone metastases [82,83,84,85,86]. Activation of the RANK pathway, which promotes osteoclast differentiation, is responsible for osteolytic metastasis and subsequent SREs [87]. Importantly, SREs adversely affect both patient quality of life and OS [88,89,90,91,92], highlighting the importance of preventing SREs during the treatment of bone metastasis. RANK ligand (RANKL) is a central activator of the NF-κB pathway and can activate the NF-κB response in cancer with bone metastasis [87]; targeting the RANKL/RANK/NF-κB pathway has recently been proposed as an effective strategy in patients with bone metastases [93,94]. BTAs, including bisphosphonates and denosumab, which were developed for the treatment of osteoporosis, have also been used to treat patients with NSCLC with bone metastases based on the association of the RANKL/RANK/NF-κB pathway with tumorigenesis. Notably, bisphosphonates have been used in the management of patients with different types of cancer and bone metastases [94,95,96,97,98,99]. However, only a few studies have examined the efficacy of bisphosphonates and standard anticancer treatment in patients with NSCLC with bone metastases, and there are no clear recommendations [93,100]. Two review articles have addressed this issue and considered several factors, including potential nephrotoxicity owing to the combination of platinum-based doublet chemotherapy and bisphosphonates, and the reduced benefit in patients with NSCLC compared with other malignancies [83,97]. One meta-analysis examined the use of BTAs in patients with NSCLC with bone metastases and reported that both bisphosphonates and denosumab reduced SREs and bone pain and tended to lead to better median PFS and median OS [101]. Two clinical trials comparing denosumab to zoledronic acid in patients with lung cancer and bone metastases reported improved median OS in the denosumab group and noninferiority to zoledronic acid in terms of SREs [93,102]. However, one recent clinical trial evaluated the addition of denosumab to standard first-line therapy in patients with NSCLC with bone metastases and did not find a median OS benefit for denosumab [103]. These conflicting results have prompted further studies examining the efficacy of denosumab and other new drugs in treating patients with bone metastases. Current guidelines recommend that treatment should be initiated with BTAs, in addition to standard first-line therapy for metastatic bone disease [104].

Radiotherapy is a therapeutic option for treating patients with NSCLC with bone metastases. Studies on bone metastases, including patients with NSCLC, reported that external beam radiotherapy (EBRT) is the most effective treatment modality for pain relief [81,105,106,107], whereas a single fraction of radiotherapy was shown to be equally effective as multifractionated radiotherapy [106,107]. SBRT, another radiation modality that delivers precise and very intense doses of radiation to cancer cells while minimizing damage to normal tissues, has been shown to improve local control rates and pain relief in patients with spinal metastases with or without surgery [108,109]. One randomized trial demonstrated that SBRT provided quicker and more durable pain relief than EBRT [109]; however, there were no differences between groups regarding functional or psychological aspects of quality of life [110]. A multicenter study showed promising outcomes in lung cancer for patients treated with ablative SRT at metastatic sites including bone [111]. Data were collected from eight centers between January 2016 and January 2017, and 26 of 333 lesions were obtained from the bone. The outcomes were fair, and the toxicity was acceptable. Better outcomes were observed in patients with low disease burden and well-controlled primary tumors.

NSCLC is associated with high expression of bone morphogenic protein 2 (BMP2) [112,113,114,115]. Fei et al. demonstrated a relationship between the activation of BMP2 and metastatic bone tumors in animal models of mouse Lewis lung carcinoma [116]. BMP2 was also shown to be associated with poor outcomes, including migration and invasion, in NSCLC cells. The BMP2 signaling pathway is associated with osteolytic and osteoblastic activities, which are strongly involved in bone metastasis. Accordingly, inhibition of BMP2 signaling may have applications in preventing NSCLC with bone metastases.

## 5. Lung Oligometastases

In NSCLC, the most common metastatic sites are the bones and lungs, whereas metastases to the liver and brain are more common in patients with small cell lung cancer [117]. Tentative staging after a comprehensive analysis of clinicopathological characteristics plays an important role in predicting long-term OS. According to the current TNM staging system, disease with pleural metastasis or contralateral lung metastases in the absence of extrathoracic metastasis is defined as M1a. Intrapulmonary metastasis in the same lobe or different ipsilateral lobes is defined as T3 or T4, respectively [118]. Owing to advancements in imaging techniques, more subtle pulmonary lesions can be detected during staging. However, the possible presence of different entities can complicate the diagnosis. For example, patients with bilateral lung lesions can be categorized as having separate primary lung cancers or one primary lung cancer with lung-to-lung metastasis, which is defined as M1a. Likewise, patients with unilateral lung lesions can also be categorized as having separate primary lung cancers or one primary lung cancer with intrapulmonary metastasis, which is defined as T3 or T4. In the pre-molecular biology era, the differential diagnosis of multiple pulmonary lesions depended on imaging studies and the personal experience of the physician. Thus, it was possible to undertreat patients with multiple pulmonary lesions if the lesions were considered to be advanced-stage NSCLC rather than separate primary lung cancers. The ambiguous diagnoses of recruited patients in previous studies also make it difficult to determine the specific effects of treatment. Synchronous multiple primary lung cancers (SMPLCs) are defined as coexisting primary lung cancers detected simultaneously. Multiple studies have reported surgical outcomes in patients with SMPLC; however, the researchers in all of these studies recruited mixed populations, including both true multiple primary lung cancer and primary lung cancer with intrapulmonary metastases [119,120,121,122]. The promising surgical outcomes from these studies may reflect the success of the treatment strategy for multiple primary lung cancers rather than the effects of local ablative therapy on NSCLC with lung oligometastases. Notably, the diagnostic criteria commonly used to differentiate SMPLC from intrapulmonary metastases do not actually yield accurate diagnoses [37,123]. The molecular profiling of tumors thus may help to guide SMPLC diagnoses and could highlight the necessity for resection of all intrapulmonary lesions to achieve accurate staging and personalized management. Additional well-designed studies are needed to clarify the role of local ablative therapy in patients with NSCLC with lung oligometastases.

In addition to surgical resection, locally ablative therapies are now commonly integrated into combined treatment strategies. Owing to advancements in techniques, high-dose radiotherapy, including SBRT or stereotactic ablative radiation therapy (SABR), delivered in up to five treatments, is a current treatment modality for patients with oligometastatic NSCLC [124]. De Rose et al. reported the outcomes of applying SABR in patients with NSCLC with lung oligometastases. Their results showed that as the diameter of the lesion increases, the radiation dose and fraction should be adjusted. Among 90 total lesions from 60 patients with NSCLC, the local control rate at 2 years was 88.9%, and the OS rates at 1 and 2 years were 94.5% and 74.6%, respectively. Notably, no pulmonary toxicity was reported [125]. However, for lesions located centrally, defined as being within 2 cm from midline structures, such as the bronchial tree, heart, great vessels, and other mediastinal organs, the adverse effects of radiation therapy were high. Some life-threatening conditions, such as hemorrhage or broncho-esophageal fistula have been reported in patients receiving a dose of approximately 60 Gy in 12 fractions [126,127,128]. Mauro et al. reported the application of SABR to ultra-central lung oligometastases in NSCLC at a relatively high dose. The fatal adverse effect was acceptable for a median BED of 105 Gy, and the outcome was superior at doses greater than 75 Gy. The delivery of ablation therapy to central metastases avoids local progression and may alter the nature of the disease [128]. In addition, LCT has shown promising outcomes. For example, Mitchell et al. reported a clinical trial of 194 patients with less than four synchronous metastatic NSCLC lesions including lung; comprehensive LCT was shown to be an independent factor improving median OS, and patients without thoracic nodal disease, bone metastases, or more than 1 metastatic site were found to have superior outcomes [129].

## 6. Liver Oligometastases

Approximately 3.8–31% of patients with newly diagnosed stage IV NSCLC have liver metastases [130,131,132]. The presence of liver metastases often represents unfavorable prognosis and treatment-resistant condition [131,133]. Due to the relatively low incidence of NSCLC with metastases other than brain, bone and adrenal gland, few articles addressed the issue about LCT for the treatment of extra-cranial, extra-skeletal and extra-adrenal NSCLC oligometastases. The first systemic review examined the efficacy of metastatectomy for extra-cranial extra-adrenal NSCLC solitary metastases, which reported a 5-year survival rate of 50% and indicated that mediastinal lymph node involvement was a poor prognostic factor [134]. The study highlighted the possibility of long-term survival benefit in selected NSCLC patients with extra-cranial extra-adrenal oligometastases following metastatectomy. In line with the study, one original article also demonstrated the importance of nodal stage as a prognostic factor and the survival benefit of local ablative treatments in selected advanced-stage NSCLC patients with median OS of 35.2 months [135]. However, the efficacy of LCT for the treatment of NSCLC patients with liver oligometastases could not be drawn from the two studies due to relatively small sample size and various clinical outcomes.

The anatomical and functional characteristics of liver metastases such as the involvement of great vessels, tumor size, bleeding risk and functional reserve limit the feasibility of surgical intervention [136]. Thus, SBRT has become an option in NSCLC patients with liver oligometastases who are unsuitable for surgery. There are several retrospective studies evaluating the efficacy of SBRT in patients with liver oligometastases from different origins, with reported 1-year survival rate ranging from 62% to 80% [137,138,139,140]. These preliminary studies demonstrated the clinical benefit of SBRT on patients with liver oligometastases with acceptable toxicity profile; however, future clinical trials are needed to examine the efficacy of LCT including surgery or SBRT specifically in patients with NSCLC with liver oligometastases.

## 7. Conclusions

With advancements in diagnostic tools and the establishment of screening programs, the incidence of oligometastatic NSCLC is expected to increase. The prognosis of patients with oligometastatic NSCLC is not as poor as traditionally believed, and the disease can be treated with curative-intent management to improve PFS and OS. Surgery or stereotactic radiosurgery can be applied as local ablative therapy and should be instituted for patients with oligometastatic NSCLC exhibiting stable disease after systemic treatment rather than maintenance therapy or observation alone. Although few studies have directly compared surgery with stereotactic radiosurgery for the treatment of oligometastases, each modality should be regarded as effective management, and the choice of local ablative therapy should be individualized after consideration of multiple factors. Numerous ongoing trials have been conducted to examine the clinical outcomes of local ablative therapy in primary lung lesions and different sites of oligometastases, particularly in the era of targeted therapy. Further studies of the efficacy of local ablative therapy in patients with NSCLC with different oligometastatic sites are expected to be published in upcoming years. Additionally, the introduction of site-specific therapies, such as bone-targeted agents, also highlights that patient quality of life should also be a major concern during the treatment of oligometastases. Establishment of a uniform definition of oligometastases and advancements in local ablative therapy will necessitate more well-designed studies to consolidate evidence in the field of oligometastatic NSCLC and provide patients with more comprehensive and effective management strategies.

## Figures and Tables

**Table 1:** Ongoing trials of interventions for NSCLC patients with brain oligometastases (https://clinicaltrials.gov).

Identifier (ClinicalTrials.gov)	Study Title	Objectives	Estimated Primary Completion Date	Primary Outcome	Phase
NCT04787185	Stereotactic Radiotherapy in Association with Immunotherapy for the Treatment of NSCLC Brain Metastases (STRAITLUC)	Examine the feasibility and intracranial control of the disease in patients with NSCLC with up to 10 brain metastases who are candidates for SRS during the course of immunotherapy	1 December 2021	Proportion of patients who experience grades 3–5 toxicity within 3 months of initiation	N/A
NCT02831959	Pivotal, Open-label, Randomized Study of Radiosurgery with or Without Tumor Treating Fields (TTFields) for 1–10 Brain Metastases From NSCLC	Examine efficacy and safety outcomes in patients with NSCLC following SRS for up to 10 brain metastases, treated with TTFields compared with supportive care alone	September 2022	Time to intracranial progression	III
NCT04058704	A Study to Determine the Efficiency for Brain Metastasis NSCLC Patients Treated with Icotinib Alone or Combined with Radiation Therapy (SMART)	Examine the optimal timing of radiotherapy for patients with NSCLC with brain metastases and *EGFR* mutation	31 December 2022	Overall survival	III
NCT02726568	High Dose Icotinib With Sequential SRS For NSCLC Patients Harboring *EGFR* Mutation with Brain Metastases	Examine the efficacy of combined high-dose icotinib and SRS for patients with NSCLC with up to 10 brain metastases	December 2021	Intracranial progression-free survival	II
NCT03769103	Study of Osimertinib + SRS vs. Osimertinib Alone for Brain Metastases in *EGFR* Positive Patients With NSCLC	Evaluate the efficacy of osimertinib alone for brain metastases compared with SRS and osimertinib in patients with NSCLC with up to 10 brain metastases and *EGFR* mutation	April 2025	Intracranial progression-free survival	II
NCT05021328	Toripalimab Combined with Anlotinib and SBRT in Patients with Untreated Brain Metastases of Driven Gene-negative NSCLC	Evaluate the efficacy and safety of toripalimab combined with anlotinib and SRS for patients with NSCLC with up to 5 brain metastases and nondriver gene mutation	1 October 2022	Intracranial response rate (iORR)	I
NCT04905550	Almonertinib Combined with Cerebral Radiation Treat Brain Metastases from *EGFR* Positive NSCLC	Evaluate the efficacy of early SRS combined with almonertinib in patients with *EGFR*-positive NSCLC with up to 4 brain metastases	1 March 2024	Intracranial progress free survival (iPFS)	II
NCT04291092	Camrelizumab Combined with Local Treatment in NSCLC Patients with Brain Metastases	Evaluate the efficacy of camrelizumab combined with chemotherapy and local treatment in NSCLC with brain metastases	30 June 2022	6 month progression-free survival rate	II
NCT03497767	A Randomised Phase II Trial of Osimertinib With or Without SRS for *EGFR* Mutated NSCLC with Brain Metastases (OUTRUN)	Evaluate the efficacy of osimertinib alone versus SRS plus osimertinib on intracranial disease control in patients with NSCLC with up to 10 brain metastases and *EGFR* mutation	August 2023	Intracranial progression free survival at 12 months	II
NCT04345146	Bevacizumab Combined with Fractionated Stereotactic Radiotherapy for 1 to 10 Brain Metastases from Non-squamous NSCLC	Evaluate the efficacy of bevacizumab combined with fractionated stereotactic radiation therapy (FSRT) in patients with NSCLC with up to 10 brain metastases	31 December 2021	Objective response rate	II
NCT03801200	Apatinib Combined with Radiotherapy in Patient with Brain Metastases from Drive Gene Negative NSCLC	Evaluate the efficacy of concurrent radiotherapy with apatinib in patients with brain metastases from drive gene wide-type NSCLC	10 September 2021	Intracranial progression-free survival (iPFS)	II
NCT04180501	SRS Sequential Sintilimab in Brain Metastasis of NSLSC	Evaluate the efficacy of SRS sequential sintilimab in patients with NSCLC with brain metastases	September 2021	Intracranial progression-free survival (iPFS)	II
NCT02858869	Pembrolizumab and Stereotactic Radiosurgery for Melanoma or Non-small-cell Lung Cancer Brain Metastases	Evaluate the side effects of pembrolizumab together with SRS for patients with NSCLC with up to 10 brain metastases	19 November 2020	Proportion of dose-limiting toxicities defined as Radiation Therapy Oncology Group grade 3 central nervous system toxicities	I
NCT04147728	Stereotactic Radiosurgery Combination with Anlotinib for Limited Brain Metastases with Perilesional Edema in NSCLC	Determine whether SRS combined with anlotinib is effective in the treatment of patients with NSCLC with up to 5 brain metastases	15 December 2021	Edema index	II

N/A = not applicable; NSCLC = non-small-cell lung cancer; SRS = stereotactic radiosurgery; *EGFR* = epidermal growth factor receptor.

**Table 2:** Ongoing trials of SBRT in patients with NSCLC (https://clinicaltrials.gov).

Identifier (ClinicalTrials.Gov)	Study Title	Objectives	Estimated Primary Completion Date	Primary Outcome	Phase
NCT02314364	A Phase II Trial of Integrating Stereotactic Body Radiation Therapy with Selective Targeted Therapy in Stage IV Oncogene-driven Non-small-cell Lung Cancer	The investigational intervention in this study was SBRT with proton or photon radiation. “Investigational” indicates that the intervention is being studied. SBRT and proton radiation therapy are FDA-approved radiation delivery systems. However, using SBRT as a treatment for stage IV NSCLC is still investigational.	November 2021	To analyze the frequency of patients with DF (with or without concurrent original site failure) in patients with oncogene-driven NSCLC with residual oligometastatic disease at 12 months after initiation of SBRT	II
NCT04306926	An Open-label, Single-arm, Phase II Study of TQB2450 Injection Combined with Stereotactic Body Radiation Therapy (SBRT) in Subjects with Advanced Oligometastatic Non-small-cell Lung Cancer	Examine efficacy and safety outcomes in patients with NSCLC injected with a humanized monoclonal antibody targeting programmed death ligand-1 (PD-L1) combined with SBRT.	30 May 2023	Progression-free survival (PFS) from baseline to 96 weeks	II
NCT03275597	Phase Ib Study of Stereotactic Body Radiotherapy (SBRT) in Oligometastatic Non-small Lung Cancer (NSCLC) With Dual Immune Checkpoint Inhibition	Examine the sequential delivery of SBRT to all disease sites followed by combination treatment with durvalumab and tremelimumab for patients for whom the goal is ablating all known sites of disease	July 2022	Safety and tolerability of SBRT followed by combined durvalumab and tremelimumab, assessed by CTCAE v4.03	I
NCT04517526	Efficacy and Safety of Platinum-based Chemotherapy + Bevacizumab + Durvalumab, and Salvage SBRT for IV Non-small-cell Lung Cancer Patients with *EGFR* Mutations After Failure of First Line Osimertinib: A Multicenter, Prospective, Phase II Clinical Study	Examine the efficacy of combined platinum-containing dual-drug chemotherapy + bevacizumab + SBRT/SRT for *EGFR*-mutant non-small-cell lung cancer with first-line progression of osimertinib, taking into account the current clinical research status	1 November 2022	PFS and overall survival (OS) within 2 years	II
NCT03808337	A Phase II Randomized Study Assessing the Efficacy of Stereotactic Body Radiotherapy (SBRT) in Patients with Oligometastatic Breast or Lung Cancer	To determine whether SBRT, when delivered to all sites of disease in participants with 1–5 metastases, will increase the length of time before disease progression.	January 2022	PFS	II
NCT02759783	A Randomised Trial of Conventional Care Versus Radioablation (Stereotactic Body Radiotherapy) for Extracranial Oligometastases	A randomized controlled trial of SBRT in patients with cancer in one of three primary sites where oligometastatic disease relapse is a common clinical scenario (breast, prostate, and NSCLC).	October 2024	PFS	II
NCT03965468	A Multicentre Single Arm Phase II Trial Assessing the Efficacy of Immunotherapy, Chemotherapy and Stereotactic Radiotherapy to Metastases Followed by Definitive Surgery or Radiotherapy to the Primary Tumour, in Patients with Synchronous Oligo-metastatic Non-small-cell Lung Cancer	Assessing the efficacy of immunotherapy, chemotherapy plus stereotactic radiotherapy for metastases followed by definitive surgery or radiotherapy to the locoregional primary tumor, in patients with histologically confirmed synchronous oligometastatic NSCLC.	December 2021	PFS at 12 months	II
NCT03808662	Precision Radiation for OligoMetastatIc and MetaStatic DiseasE (PROMISE)-004: Consolidative Use of Radiotherapy to Block (CURB) Oligoprogression	Determine whether receiving SBRT when participants’ metastatic tumors have just begun to grow increases the length of time before disease progression	January 2022	PFS	II
NCT03497767	A Multicentre Single-arm Phase II Trial Assessing the Safety and Efficacy of First-line Osimertinib and Locally Ablative Radiotherapy in Patients with Synchronous Oligo-metastatic *EGFR*-mutant Non-small-cell Lung Cancer	Evaluate the safety and efficacy of osimertinib combined with early locally ablative radiotherapy of all cancer sites in patients with synchronous oligo-metastatic (primary tumor and maximum 5 metastases) *EGFR*-mutant (exon 19 deletion or exon 21 L858R) NSCLC	March 2026	Safety and efficacy of first-line *EGFR*-targeting osimertinib and SBRT to the primary tumor and all metastases	II

SBRT = stereotactic body radiation therapy; DF = distant failures; NSCLC = non-small-cell lung cancer; FDA = Food and Drug Administration; CTCAE = common terminology criteria for adverse events; *EGFR* = epidermal growth factor receptor.

## Data Availability

No new data were created or analyzed in this study. Data sharing is not applicable to this article.
